# 307. Diagnostic Test Kinetics, Infectivity, and Immunological Responses Among Unvaccinated Adults During Acute SARS-CoV-2 Infection

**DOI:** 10.1093/ofid/ofac492.385

**Published:** 2022-12-15

**Authors:** Paul K Drain, Ronit Dalmat, Meagan Bemer, Elvira Budiawan, Jennifer F Morton, Linhui Hao, Renee Ireton, Zarna Marfatia, Adanech Gichamo, Roshni Prabhu, Claire Woosley, Elena A Rechkina, Daphne Hamilton, Michalina A Montano, Jason L Cantera, Inah D Golez, Elise Smith, Alexander L Greninger, Benjamin D Grant, Allison Meisner, Geoffrey S Gottlieb, Michael Gale

**Affiliations:** University of Washington, Seattle, Washington; University of Washington, Seattle, Washington; University of Washington, Seattle, Washington; University of Washington, Seattle, Washington; University of Washington, Seattle, Washington; University of Washington, Seattle, Washington; University of Washington, Seattle, Washington; University of Washington, Seattle, Washington; Wellstar Atlanta Medical Center, Atlanta, Georgia; University of Washington, Seattle, Washington; University of Washington, Seattle, Washington; International Clinical Research Center, Department of Global Health, University of Washington, Seattle, Washington; University of Washington, Seattle, Washington; University of Washington, Seattle, Washington; Global Health Labs, Bellevue, Washington; University of Washington, Seattle, Washington; University of Washington, Seattle, Washington; University of Washington, Seattle, Washington; Global Health Labs, Bellevue, Washington; Fred Hutchinson Cancer Center, Seattle, Washington; University of Washington, Seattle, Washington; University of Washington, Seattle, Washington

## Abstract

**Background:**

Appropriate diagnostic testing can be used to inform infection control measures and reduce SARS-CoV-2 transmission, yet the test kinetics, infectivity, and immunological responses during acute, non-severe SARS-CoV-2 infection need clarity.

**Methods:**

We conducted a prospective cohort study between Nov 2020-July 2021 in Seattle, Washington of 95 unvaccinated, immunocompetent adults with no prior SARS-CoV-2 infection. Nasal swabs (nasopharyngeal and anterior) and blood serum samples were serially collected at six visits over two months. Viral RNA, N and S antigen concentrations, and viral growth/infectivity were measured from nasal samples. Anti-S total antibody and IgG assays were performed on serum. We fit loess curves to quantitative data corresponding to each testing modality by days since symptom onset (DSSO) and compared qualitative test results across time points to demonstrate time-dependent agreement of PCR, N antigen, and culture results. Generalized estimating equations were used to approximate relative risk of culture positivity (a proxy for infectiousness) for positive vs. negative test results (antigen and PCR), stratified by presence/absence of symptoms and DSSO.

Sampling Schema

**Figure:**
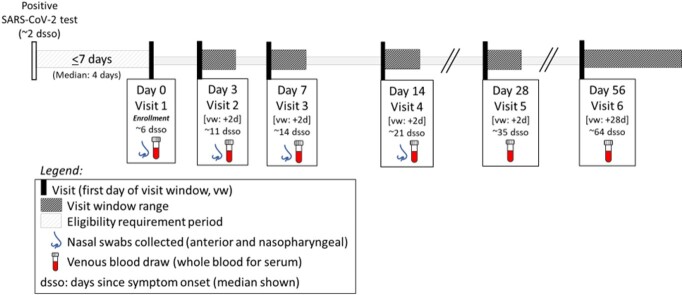

Nasal swabs and venous blood were collected at visits 1-4; venous blood only at visits 5-6. All participants were enrolled within 14 days of symptom onset (median: 6) and 7 days of a positive test (median: 4).

**Results:**

Infections in this cohort (median age: 29y) were mild (no hospitalization). Median (IQR) time to negative result was 11 (4), 13 (6), and 20 (7) DSSO for culture growth, N antigen, and PCR tests, respectively. Viral RNA quantities declined more slowly than antigen and culturable virus; antibody titers rose rapidly 5-15 DSSO and plateaued 20-30 DSSO. All culture-positive samples collected 0-5 DSSO were positive by PCR, but relative risk of culture positivity (infectiousness) for positive vs. negative PCR results declined 6-10 DSSO. Relative risk of culture positivity for positive vs. negative antigen results was consistently high 0-10 DSSO, with similar results when stratified by presence of symptoms.

Diagnostic test kinetics and immunological responses

**Figure:**
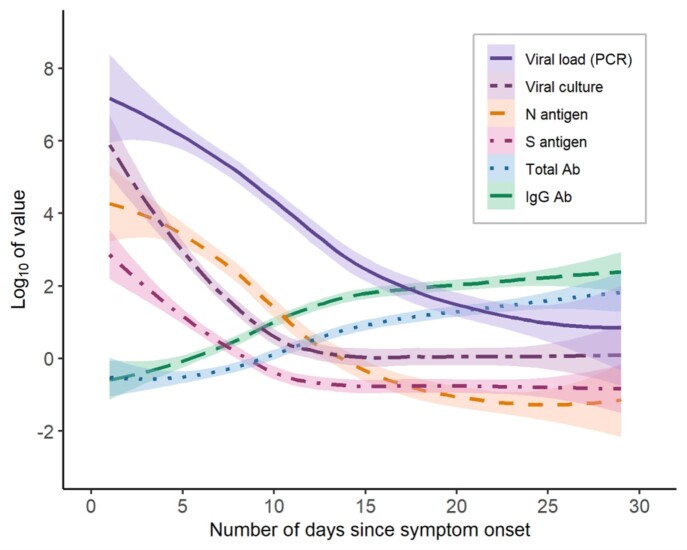

Diagnostic test kinetics and immunological responses measured in adults with non-severe, symptomatic SARS-CoV-2 infection: loess trendlines and 95% confidence intervals are given for SARS-CoV-2 viral load (calculated from PCR Ct value using a calibration curve), TCID50 from viral culture, mean concentrations of nucleocapsid and spike antigen proteins, and anti-S total and IgG antibody concentrations.

**Conclusion:**

The results reinforce the importance of molecular PCR testing as a highly sensitive diagnostic tool but with limited utility as an indicator of viral culturability and likely infectiousness. N antigen testing may be a preferable diagnostic test within two weeks of symptom onset, especially 6-10 DSSO, because it more closely correlates with culture growth over the course of infection.

**Disclosures:**

**Daphne Hamilton, BA**, Roche (spouse is employed by Roche): Employee **Alexander L. Greninger, MD, PhD**, Abbott: Contract Testing|Cepheid: Contract Testing|Gilead: Grant/Research Support|Gilead: Contract Testing|Hologic: Contract Testing|Merck: Grant/Research Support|Novavax: Contract Testing|Pfizer: Contract Testing **Geoffrey S. Gottlieb, MD, PhD**, Abbott Molecular Diagnostics: Grant/Research Support|Alere Technologies: Grant/Research Support|BMGF: Grant/Research Support|BMS: Grant/Research Support|Cerus Corp.: Grant/Research Support|Gilead Sciences: Grant/Research Support|Janssen Pharmaceutica: Grant/Research Support|Merck & Co: Grant/Research Support|Roche Molecular Systems: Grant/Research Support|THERA Technologies/TaiMed Biologics: Grant/Research Support|ViiV Healthcare: Grant/Research Support.

